# Using Mobile Health to Enhance Outcomes of Noncommunicable Diseases Care in Rural Settings and Refugee Camps: Randomized Controlled Trial

**DOI:** 10.2196/mhealth.8146

**Published:** 2018-07-13

**Authors:** Shadi Saleh, Angie Farah, Hani Dimassi, Nour El Arnaout, Joanne Constantin, Mona Osman, Christo El Morr, Mohamad Alameddine

**Affiliations:** ^1^ Health Management and Policy Faculty of Health Sciences American University of Beirut Beirut Lebanon; ^2^ Global Health Institute American University of Beirut Beirut Lebanon; ^3^ School of Pharmacy Lebanese American University Beirut Lebanon; ^4^ Department of Family Medicine Faculty of Medicine American University of Beirut Beirut Lebanon; ^5^ School of Health Policy and Management Faculty of Health York University Toronto, ON Canada; ^6^ Health Management and Policy College of Medicine Mohammed bin Rashid University of Medicine and Health Sciences Dubai United Arab Emirates

**Keywords:** noncommunicable diseases, hypertension, diabetes mellitus, telemedicine, mobile health, rural health, refugees

## Abstract

**Background:**

Rural areas and refugee camps are characterized by poor access of patients to needed noncommunicable disease (NCD)–related health services, including diabetes and hypertension. Employing low-cost innovative eHealth interventions, such as mobile health (mHealth), may help improve NCDs prevention and control among disadvantaged populations.

**Objective:**

The aim of this study was to assess the effect of employing low-cost mHealth tools on the accessibility to health services and improvement of health indicators of individuals with NCDs in rural areas and refugee camps in Lebanon.

**Methods:**

This is a randomized controlled trial study in which centers were allocated randomly into control and intervention sites. The effect of an employed mHealth intervention is assessed through selected quality indicators examined in both control and intervention groups. Sixteen primary health care centers (eight controls, eight interventions) located in rural areas and Palestinian refugee camps across Lebanon were included in this study. Data on diabetic and hypertensive patients—1433 in the intervention group and 926 in the control group—was extracted from patient files in the pre and postintervention periods. The intervention entailed weekly short message service messages, including medical information, importance of compliance, and reminders of appointments or regular physician follow-up. Internationally established care indicators were utilized in this study. Descriptive analysis of baseline characteristics of participants, bivariate analysis, logistic and linear regression were conducted using SPSS (IBM Corp).

**Results:**

Bivariate analysis of quality indicators indicated that the intervention group had a significant increase in blood pressure control (*P*=.03), as well as a significant decrease in the mean systolic blood pressure (*P*=.02), mean glycated hemoglobin (HbA_1c_; *P*<.01), and in the proportion of HbA_1c_ poor control (*P*=.02). Separate regression models controlling for age, gender, and setting showed a 28% increase in the odds of blood pressure control (*P*=.05) and a 38% decrease in the odds of HbA_1c_ poor control (*P*=.04) among the intervention group in the posttest period. Females were at lower odds of HbA_1c_ poor control (*P*=.01), and age was statistically associated with annual HbA_1c_ testing (*P*<.01). Regression models for mean systolic blood pressure, mean diastolic blood pressure, and mean HbA_1c_ showed that a mean decrease in HbA_1c_ of 0.87% (*P*<.01) pretest to posttest period was observed among the intervention group. Patients in rural areas belonging to the intervention group had a lower HbA_1c_ score as compared with those in refugee camps (*P*<.01).

**Conclusions:**

This study underlines the importance of employing integrative approaches of diseases prevention and control in which existing NCD programs in underserved communities (ie, rural and refugee camps settings) are coupled with innovative, low-cost approaches such as mHealth to provide an effective and amplified effect of traditional NCD-targeted care that can be reflected by improved NCD-related health indicators among the population.

**Trial Registration:**

ClinicalTrials.gov NCT03580330; https://clinicaltrials.gov/ct2/show/NCT03580330 (Archived by WebCite at http://www.webcitation.org/70mhVEUwQ)

## Introduction

### Prevalence of Noncommunicable Diseases

Noncommunicable diseases (NCDs) and its associated risk factors, including diabetes mellitus and hypertension (HTN), have become the leading cause of death and disability globally [1]. In 2015, NCDs accounted for 70% of the estimated 56.4 million deaths in the world [2]. Around 80% of deaths associated with NCDs occur in low- and middle-income countries (LMIC), with disproportional effects on underprivileged populations [3-5].

In the Eastern Mediterranean Region (EMR), the burden of NCDs is growing at an alarming rate, with approximately 57% of deaths in the region attributed to NCDs, an equivalent of 2.2 million death per year [6]. Around 51% of these deaths are premature (ie, below the age of 70 years) [6-8]. Unhealthy diets, physical inactivity, hyperglycemia, hyperlipidemia, elevated blood pressure (BP), and obesity are among the most prevalent underlying risk factors; linked to 65% of NCDs-related deaths [7,9,10].

### Limited Access to Health Care in Rural Settings and Refugee Camps

Despite the rising burden of NCDs, health care systems of the majority of LMIC remain focused on treatment with minimal unsustainable investment in primary health care (PHC) [11]. Different economic, sociocultural, and geographic factors were also found to limit access of patients to NCDs preventive health care services in these settings [12,13]. Lack of essential knowledge and awareness on NCDs prevention, especially among underserved populations, often results in poor management of their disease, manifested in substandard health-related indicators manifested in poor glycemic or BP control and other preventable morbidities [14-22]. For refugees in specific, the situation is further aggravated by their restricted access to health care services because of a range of factors, including financial barriers, geographic attainability, safety, as well as cultural and language impediments [14]. NCD-specific care requires a systematic approach ranging from case finding and early detection, to identification of unhealthy behaviors, and compliance to regular long-term follow-up [23]. Such an approach is constrained by the limited health facility-based resources in rural and refugee settings, which hinders the ability to implement proper NCD preventive measures in those settings. Further compounding the situation is the ongoing displacement of Palestinian and Syrian refugees and the protracted crises in the EMR, adding burden to already fragmented health systems of refugee-hosting countries regionally [7,18].

### Use of Mobile Health as an Effective Add-On to Traditional Care

Despite the fact that remarkable efforts have been invested to decrease the burden of NCDs in EMR [21,22], a comprehensive change in the approach to NCDs in these settings remains necessary to meet the health needs of the displaced populations and host communities [14,16,20]. A systematic review of primary care models for NCDs interventions in LMIC recommends a programmatic structure focused on monitoring and evaluation of indicators, standardized care, and compliance to follow-up [24].

With the spread of mobile technology, mobile Health (mHealth) is regarded as a promising approach that is being increasingly explored to improve community health outcomes. As a subset of electronic health (eHealth), mHealth is defined as the use of mobile devices in health care delivery, mainly through short message service (SMS) messaging, voice calls, mobile phone apps, tablets, or wearable devices’ apps [25]. SMS is an mHealth tool that holds great promise in addressing NCDs through health education and self-management, improving prevention and treatment strategies, and providing appointment reminders to improve compliance and the attendance of appointments in PHC centers (PHCCs). SMSs are also attractive because of their potential in overcoming financial and geographic barriers facing hard-to-reach populations [26-33]. A number of academic studies showed that mHealth helps improve prevention and control of diseases, including HTN and diabetes, by providing targeted interventions to disadvantaged populations living in remote areas where health services are often limited [26,34-43].

### Lebanese Context and Relevance of the Study

According to recent reports, Lebanon faces an elevated NCDs-related mortality reaching as high as 85% [44]. Furthermore, the country hosts a large proportion of the world’s Palestinian refugees, accounting around 10% of the country’s population [45]. The burden of NCDs, namely diabetes and HTN, is expanding among underprivileged populations residing in rural areas and refugee camps primarily because of poor screening and low early detection rates [46]. Suffering from limited availability of resources and fragmented infrastructure, PHCCs are to a large extent the sole convenient health facilities serving people in Lebanese rural areas where most refugees are residing [47]. The 2011 assessments of the United Nations Relief and Works Agency (UNRWA) revealed that one-third of Palestinian refugees in Lebanon residing in camps face hardships related to NCDs [48]. The refugees’ struggle is aggravated by the unsatisfactory health services in such contexts [49]. Exploring innovative and effective strategies that can complement existing traditional care remains necessary in such settings to appropriately tackle the growing trend of NCDs in the country.

Mobile phone use is very common in Lebanon, reaching as high as 92.16% in 2015 [50]. A situational assessment study on Syrian refugees and digital health in Lebanon reported refugees as frequent users of mobile communications, including SMS, with each household having at least one mobile phone, suggesting the likely reach that an mHealth intervention could have in this context [51].

Despite the proven abundance of mobile phones in underserved settings, the potential role that mHealth could have in enhancing access of disadvantaged populations to adequate NCD care has never been investigated in Lebanon.

This study aims to assess the effect of employing a low-cost mHealth intervention on access to health services and improvement of health indicators of individuals suffering from NCDs in rural areas and refugee camps in Lebanon. The mHealth intervention is of dual components addressing both patients and health providers through informative health SMSs targeting the former and online training modules and support forums targeting the latter. This study, implemented in collaboration with the Lebanese Ministry of Public Health (MOPH) and UNRWA, is the first to employ a mHealth intervention in Lebanon in rural and refugee settings. The results will provide evidence that could help in restructuring existing NCDs action plan at the systems level to optimally respond to the needs of displaced refugees and host communities and enhance compliance to adequate care while containing cost through adopting an innovative preventive approach.

## Methods

### Study Design

**Figure 1:**
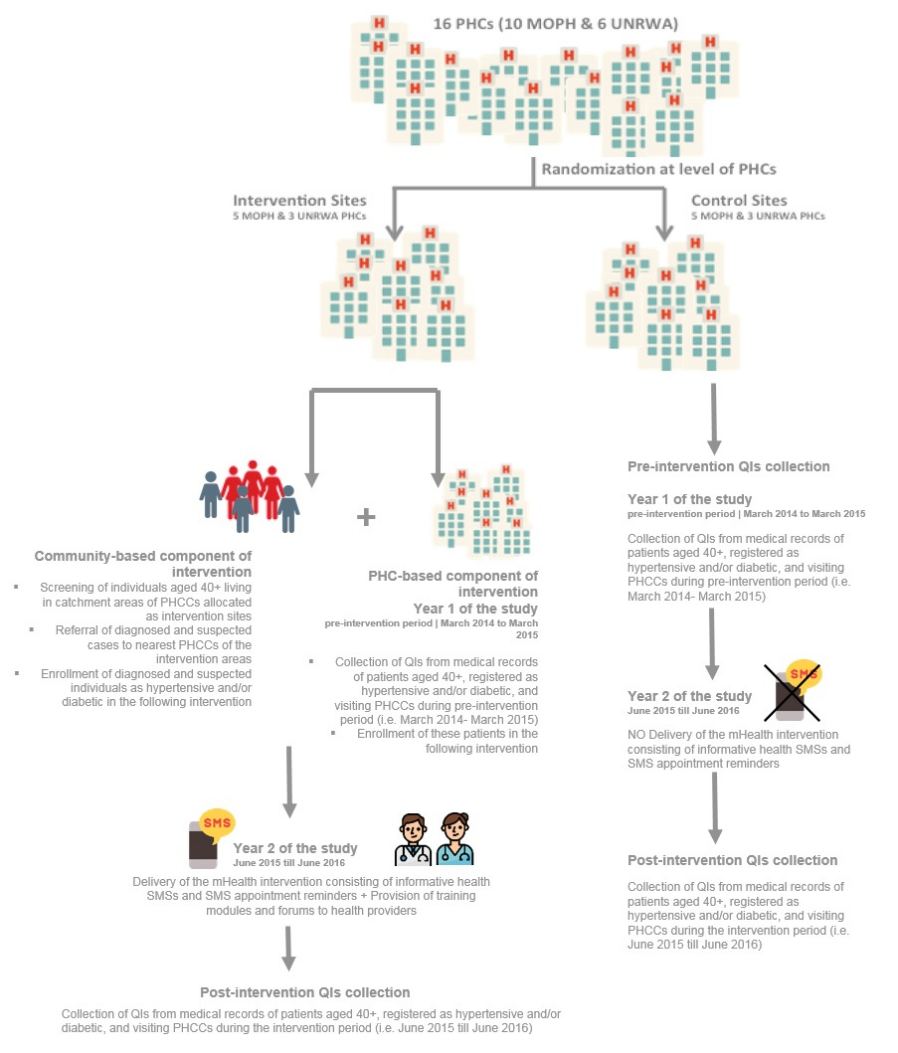
Summary of the methodology of the study. MOPH: Ministry of Public Health; PHC: primary health care; PHCC: primary health care center; QI: quality indicator; SMS: short message service; UNRWA: United Nations Relief and Works Agency.

This study reports on a community trial in which PHC centers, along their respective catchment areas, were randomly allocated into control and intervention sites with the aim of assessing the change in selected NCD care quality indicators (QIs) among community individuals and patients. Patients in the intervention sites received a 1-year mHealth intervention, and their pre- and postintervention outcomes were assessed through measurement of QIs. The study was conducted over a period of 2 years, covering 1 year of preintervention collection of QIs and 1 year of delivery of the mHealth intervention, followed by a postintervention period of 1 month of postintervention QIs collection (Figure 1).

### Participants Selection and Data Collection

The study population comprised sixteen PHCCs in Lebanon: ten located in rural areas and belonging to the Lebanese MOPH PHC National Network [52] and six are UNRWA centers chosen from Palestinian refugee camps in Lebanon. These centers were randomly assigned into intervention and control groups. Five MOPH and three UNRWA centers were allocated to each of the intervention and control groups for a total of eight sites in each of the groups.

One QI collector was hired at each of the sixteen PHCCs included in the study (both intervention and control) to collect relevant QIs from patients’ records at two points in time: (1) at baseline period, also noted as the preintervention period, where QIs were collected from records of all patients visiting included PHCCs for the 1-year preintervention period from March 2014 to March 2015 and (2) after delivery of intervention, also noted as the postintervention period, where QIs were collected from records of patients visiting the PHCCs for the 1-year intervention period from June 2015 to June 2016.

The inclusion criteria of the records of patients during the QIs collection periods was based on the health status and age of the patients. To be included in this study, patients had to be registered at the PHCCs as diabetics or hypertensive and aged 40 years or more. Only Lebanese patients registered at the included MOPH PHCCs in rural areas and Palestinian refugee patients registered at the included UNRWA health centers were eligible for inclusion if the aforementioned criteria were met. Records of patients whose nationality was not Lebanese nor Palestinian and whose age was less than 40 years were not eligible for inclusion. No exclusion based on gender, educational and literacy level, disability, or presence of other medical conditions took place. Data were extracted from patients’ medical records for all patients meeting the inclusion criteria. Demographics including gender, age, and telephone number, in addition to medical information related to the QIs such as last result of BP, last result of glycated hemoglobin (HbA_1c_), smoking status, and dates of last visit for HbA_1c_ testing, for eye check-up, or foot exam, were collected. Numerical values and dates were reported whenever available within the corresponding data collection period; otherwise, not reported was entered for missing values.

### Intervention

The eSahha project is a two-pronged mHealth interventional project targeting catchment areas of PHCCs located in Lebanese rural areas and Palestinian refugee camps, where access to health knowledge and health services is known to be limited.

The overall eSahha intervention consisted of two related components: one that is community-based and another that is PHC center-based. The community-based component included community screening for HTN and diabetes by trained community health workers among individuals falling within the age group at higher risk of developing NCDs—40 years or older—in the catchment areas of the eight intervention centers.

 Individuals already diagnosed with or suspected of being diabetic, hypertensive, or both were referred to the nearest intervention PHCC for NCD-specific clinical care and were targeted by SMS messages originating from a preexisting mobile communication platform used for mass messaging hosted by a Lebanese telecommunication company and scheduled for delivery by a research assistant of the research team. The SMSs were developed by a family physician based on the MOPH guidelines for prevention and management of HTN and diabetes. A weekly educational health SMS was sent every Monday afternoon for the intervention period of 1 year. SMS content covered different health themes providing health information on lifestyle, dietary habits, body weight, smoking, medications, importance of compliance, as well as symptoms and self-management of HTN and diabetes. Community individuals who were diagnosed and were receiving necessary care previous to our intervention were sent weekly informative health SMS, as well as customized SMSs reminders to follow up on their scheduled medical appointments (eg, to check their HbA_1c_ levels and have their annual foot or eye exams). The messages were sent to mobile phones of the targeted individuals suffering from HTN or diabetes included in the intervention or, in cases where the targeted individuals did not own a mobile phone, messages were sent with their consent to the cell phones of their respective closest relatives (eg, son, daughter, or husband). Individuals in the control group (ie, living in catchment areas of control PHCCs) did not receive SMS messages and were thus receiving the usual care. The messages were initially formulated in English and then translated to Arabic and sent using simplified Arabic terms to match the different levels of health literacy of the target lay population. The length of each message was restricted to a maximum of 70 characters. On the other hand, the PHC center-based component of the intervention consisted of sending the same weekly informative health SMS, as well as appointment reminders customized to the respective time for check-ups to patients registered as diabetic or hypertensive at baseline at the PHCCs belonging to the intervention group. Patients receiving the SMSs were not required to reply to the SMSs at any point in time.

The PHC center-based component of the intervention also included training of health care providers, namely physicians and nurses, working in the intervention PHCCs, using eHealth tools consisting of (1) Online modules focusing on clinical guidelines for treating diabetes and HTN and others on provider-patient communication strategies (ie, increasing compliance) and (2) Online forums and frequently asked questions mainly dedicated to peer-to-peer knowledge sharing of treatment and communication techniques.

### Measurements of Quality Indicators

A set of internationally recognized QIs for diabetes and HTN were employed to monitor the effectiveness of the intervention (Table 1). The choice of indicators was based on their wide acceptance and use in evaluating care effectiveness on one hand and on the ability of the health information system available in the included MOPH and UNRWA PHCCs to extract the needed data on the other hand.

### Statistical Analysis

Data on BP (systolic blood pressure, SBP or diastolic blood pressure, DBP), HbA_1c_, smoking status, as well as dates of eye and foot check-ups were obtained and analyzed using statistical software package SPSS (version 24.0). Sample baseline characteristics were summarized for the intervention and control groups using mean and SD for numerical variables and frequency and percentage for categorical variables. Annual check-ups, including eye and foot exams, as well as HbA_1c_ testing were considered acceptable if done according to recommended guideline periods, while a 30-days grace period was allowed. Pearson chi-square test (χ^2^) and independent *t* test were used to assess the difference in quality indicators before and after the intervention. Logistic regression was used to evaluate the impact of the intervention on HbA_1c_ poor control, BP control, and annual HbA_1c_ testing, while controlling for age, gender, and setting. Similarly, linear regression was used to assess the impact on mean SBP, DBP, and HbA_1c_, while controlling for age, gender, and setting. All analyses were carried at a .05 significance level.

**Table 1:** Selected quality indicators to monitor the effectiveness of the intervention.

Domain and Measure		Measure description
**Hypertension**		
	Mean blood pressure	Means SBP^a^ and DBP^b^ were assessed after collecting the most recent result of each patient’s blood pressure in terms of SBP or DBP
	Blood pressure control	Percentage of patients with most recent blood pressure <140/90 mmHg
	Annual eye check-up	Percentage of patients receiving at least one eye check-up annually as per recommended guideline (date within the data collection period, while a 30-day grace period was allowed)
**Diabetes**		
	Annual HbA_1c_^c^ testing	Percentage of patients with one or more HbA_1c_ tests annually as per recommended guideline (date within the data collection period, while a 30-day grace period was allowed)
	Mean HbA_1c_	Mean HbA_1c_ was assessed after collecting each patient’s most recent result of HbA_1c_
	HbA_1c_ poor control	Percentage of patients with most recent HbA_1c_ level >9.0% (poor control); Mean HbA_1c_ is also assessed
	Annual smoking status check	Percentage of patients whose smoking status was ascertained and documented annually
	Annual foot exam	Percentage of patients receiving at least one foot exam annually as per recommended guideline (date within the data collection period, while a 30-day grace period was allowed)
	Annual eye check-up	Percentage of patients receiving at least one eye check-up annually as per recommended guideline (date within the data collection period, while a 30-day grace period was allowed)

^a^SBP: systolic blood pressure.

^b^DBP: diastolic blood pressure.

^c^HbA_1c_: glycated hemoglobin.

### Ethical Considerations

Ethical approval was obtained from the American University of Beirut Institutional Review Board before conducting the study. As per the approved protocol, approval of participation and informed consent were obtained from PHC centers’ directors. Directors and QI collectors recruited from the centers in the two groups were informed about the purpose of the study and its expected outcomes. Confidential handling of all data collected was ensured throughout the study period. In addition, SMS messages were sent to patients in the intervention group after obtaining their consent. In case the targeted patients did not own a mobile phone, their consent was received to send the messages to the phone of their selected closest relatives (eg, son, daughter, or husband). Those relatives in turn had to also consent to receiving those messages and passing them to the patients.

## Results

### Participants’ Demographic Characteristics

Table 2 presents the baseline demographic and clinical characteristics of patients in the intervention and control groups. The data of 1433 patients in the intervention groups and 926 patients in the control groups were included in the analysis. At baseline, more than half of patients were females, 56.3% (454/807) in the intervention group and 56.2% (292/520) in the control group. As for age distribution, a higher proportion was in the age range of 56 to 70 years, representing 44.1% (353/800) of those in the intervention group and 40.1% (200/499) of those in the control. Majority of patients in both groups were living in Lebanese rural areas (61.97%, 888/1433 and 60.8%, 563/926 respectively). In the intervention group, 64.27% (921/1433) of patients were hypertensive, and 35.73% (512/1433) were diabetic. On the other hand, 67.6% (626/926) of patients in the control group were hypertensive, and 32.4% (300/926) were diabetic. There was no statistical difference between the two groups except for age (*P*=.003; Table 2).

### Hypertension Quality Indicators

Bivariate analyses of quality indicators are shown in Table 3. All dates are considered acceptable if done within the recommended guideline periods, while a 30-day grace period was allowed (refer to Table 1). A significant increase in BP control was observed in the intervention group between the pre- and the posttest periods (58.2%, 530/911 to 63.6%, 426/670, *P*=.03), which was not replicated in the control group (*P*=.37). Similarly, a significant drop in the mean SBP was observed among the intervention group (133.7 mmHg, SD 16.1 pretest and 131.8 mmHg, SD 15.8 posttest, *P*=.02) but not in the control group (*P*=.65). In both study groups, a lower proportion of annual eye check-up was observed in the post test as compared with the pretest (48.4%, 446/921 to 38.9%, 268/689 in intervention group and 27.8%, 174/626 to 21.1%, 133/630 in the control group, *P*<.01 for both). No statistical significance was found in the pre- or posttest difference in the mean DBP in either groups (*P*=.14 and *P*=.81, respectively).

**Table 2:** Baseline demographic and clinical characteristics of participants by study group. No significant differences between gender, setting, and disease category across the two groups were identified using chi-square test; the difference in age groups between intervention and control at baseline is statistically significant (*P*=.003). Bonferroni post-hoc test reveals that the difference is in the age group ≥71 years.

Variable		Intervention, n (%)	Control, n (%)
Total number of participants^a^		1433 (100.0)	926 (100.0)
**Gender**			
	Male	353 (43.7)	228 (43.8)
	Female	454 (56.3)	292 (56.2)
**Age group (years)**			
	40-55	252 (31.5)	134 (26.9)
	56-70	353 (44.1)	200 (40.1)
	≥71	195 (24.4)	165 (33.1)
**Setting**			
	Rural areas	888 (62.0)	563 (60.8)
	Palestinian refugee camps	545 (38.0)	363 (39.2)
**Disease category**			
	Diabetes	512 (35.7)	300 (32.4)
	Hypertension	921 (64.3)	626 (67.6)

^a^Some numbers under some categories may not add up to the total because of missing values.

**Table 3:** Bivariate analysis of hypertension (HTN) and diabetes mellitus quality indicators by study group.

Type of quality indicators		Intervention			Control		
		Pretest	Posttest	*P* value	Pretest	Posttest	*P* value
**HTN quality indicators**							
	BP^a^ controlled, n (%)	530 (58.2)	426 (63.6)	.03^b^	316 (60.9)	362 (58.4)	.37
	SBP^c^, mean mmHg (SD)	133.69 (16.10)	131.80 (15.79)	.02^b^	133.24 (17.08)	133.68 (16.91)	.65
	DBP^d^, mean mmHg (SD)	79.16 (9.13)	78.47 (9.09)	.14	78.24 (9.41)	78.09 (8.97)	.81
	Annual eye check-up, n (%)	446 (48.4)	268 (38.9)	<.01^b^	174 (27.8)	133 (21.1)	<.01^b^
**Diabetes mellitus quality indicators**							
	Annual HbA_1c_^e^ testing, n (%)	264 (51.6)	397 (74.1)	<.01^b^	100 (33.3)	208 (71.2)	<.01^b^
	HbA_1c_ poor control, n (%)	75 (28.2)	82 (20.3)	.02^b^	22 (21.8)	46 (22.2)	.93
	HbA_1c_, mean % (SD)	8.00 (1.89)	7.20 (2.06)	<.01^b^	7.73 (1.72)	7.69 (1.84)	.88
	Proportion of smokers, n (%)	159 (35.0)	169 (31.8)	.29	60 (36.1)	64 (31.4)	.33
	Annual foot exam, n (%)	224 (43.8)	227 (42.4)	.65	118 (39.3)	105 (36.0)	.40
	Annual eye check-up, n (%)	266 (52.0)	183 (34.1)	<.01^b^	111 (37.0)	68 (23.3)	<.01^b^

^a^BP: blood pressure.

^b^Indicates to statistical significance at .05 CI.

^c^SBP: systolic blood pressure.

^d^DBP: diastolic blood pressure.

^e^HbA_1c_: glycated hemoglobin.

### Diabetes Quality Indicators

In the intervention group, glycemic control results improved in the intervention group but not in the control group. The proportion of HbA_1c_ poor control decreased significantly in the intervention group from 28.2% to 20.3% (*P*=.02), whereas it remained unchanged in the control group (21.8% vs 22.2%, *P*=.93). Likewise, a significant reduction in the mean HbA_1c_ was noted in the intervention group (8.00%, SD 1.9 to 7.2%, SD 2.1, *P*<.01) but not in the control group (*P*=.88). Furthermore, the proportion of annual HbA_1c_ testing done within recommended guideline period increased for both groups (51.6%-74.1% in the intervention and 33.3%-71.2% in the control, *P*<.01 for both). However, the proportion of annual eye check-up done within recommended guideline period decreased significantly in both groups (52.0% down to 34.1% in intervention and 37.0% down to 23.3% in control, *P*<.01 for both). Differences in proportions of smoking and annual foot exam within recommended guideline period were not statistically significant in either group.

### Regression Models

Separate regression models were run to gauge the intervention impact on the study groups while controlling for baseline characteristics: age, gender, and setting. Table 4 shows the results of the logistic regressions, where BP control, HbA_1c_ poor control, and annual HbA_1c_ testing act as the dependent variables (consecutively). Annual HbA_1c_ testing is within recommended guideline: dates are considered acceptable if done within the recommended guideline period, while a 30-day grace period was allowed (refer to Table 1). When considering BP control, Table 4 indicates that for the intervention group, there was a 28% increase in the odds of BP control in the posttest period as compared with the pretest period (odds ratio, OR 1.28, 95% CI 1.00-1.64, *P*=.05), independent of age, gender, and setting. The OR for study period was not statistically significant in the control group (*P*=.11). Rural areas reported lower odds of BP control as compared with refugee camps in both study groups (OR 0.31, 95% CI 0.24-0.40 intervention group; OR 0.22, 95% CI 0.15-0.30 control group; *P*<.01 for both). A 38% decrease in the odds of HbA_1c_ poor control among the intervention group from the pretest to the posttest study periods was observed (OR 0.62, 95% CI 0.39-0.97; *P*=.04), independent of age, gender, and setting. The study period OR was not statistically significant among the control group (*P*=.26). Females were at lower odds of HbA_1c_ poor control among the intervention group (OR 0.59, 95% CI 0.39-0.89; *P*=.01), whereas age ORs were statistically significant for both study groups (OR 0.97 for both, *P*<.01 and *P*=.03, respectively). With regard to annual HbA_1c_ testing, the results reveal that both groups had an increase in the odds of doing the test with recommended guideline period (OR 2.52, 95% CI 1.81-3.49 for intervention and OR 4.26, 95% CI 2.79-6.49 for control; *P*<.01 for both). Age was statistically associated with annual HbA_1c_ testing (OR 0.98, *P*<.01) for the intervention group, and rural settings were at higher odds of conforming with HbA_1c_ testing guidelines in both study groups as compared with refugee camps participants (OR 4.43 for intervention and OR 2.22 for control; *P*<.01 for both).

**Table 4:** Logistic regression model of blood pressure (BP) control, glycated hemoglobin (HbA_1c_) poor control, and annual HbA_1c_ testing by study group.

Indicator			Intervention		Control	
			OR^a^ (95% CI)	*P* value	OR (95% CI)	*P* value
**BP control**						
	**Study period**					
		Posttest	1.28 (1.00-1.64)	.05^b^	1.28 (0.95-1.72)	.11
		Pretest	—^c^	—	—	—
	**Gender**					
		Female	1.12 (0.88-1.44)	.36	0.97 (0.74-1.29)	.85
		Male	—	—	—	—
	Age (continuous)		0.99 (0.98-1.00)	.06	0.99 (0.98-1.00)	.03^b^
	**Setting**					
		Rural areas	0.31 (0.24-0.40)	<.01^b^	0.22 (0.15-0.30)	<.01^b^
		Palestinian refugee camps	—	—	—	—
**HbA_1c_ poor control**						
	**Study period**					
		Posttest	0.62 (0.39-0.97)	.04^b^	0.68 (0.35-1.33)	.26
		Pretest	—	—	—	—
	**Gender**					
		Female	0.59 (0.39-0.89)	.01^b^	0.84 (0.47-1.49)	.56
		Male	—	—	—	—
	Age (continuous)		0.97 (0.96-0.99)	<.01^b^	0.97 (0.95-1.00)	.03^b^
	**Setting**					
		Rural areas	0.71 (0.45-1.11)	.13	0.81 (0.43-1.51)	.51
		Palestinian refugee camps	—	—	—	—
**Annual HbA_1c_ testing**						
	**Study period**					
		Posttest	2.52 (1.82-3.49)	<.01^b^	4.26 (2.79-6.49)	<.01^b^
		Pretest	—	—	—	—
	**Gender**					
		Female	1.17 (0.85-1.61)	.34	1.04 (0.69-1.56)	.87
		Male	—	—	—	—
	Age (continuous)		0.98 (0.97-0.99)	<.01^b^	1.01 (0.99-1.03)	.37
	**Setting**					
		Rural areas	4.43 (3.20-6.13)	<.01^b^	2.22 (1.46-3.39)	<.01^b^
		Palestinian refugee camps	—	—	—	—

^a^OR: odds ratio.

^c^Indicates to statistical significance of .05 CI.

^c^Reference category.

**Table 5:** Linear regression model of the means systolic blood pressure (SBP), diastolic blood pressure (DBP), and glycated hemoglobin (HbA_1c_) by study group.

Indicator			Intervention			Control		
			Beta	SE	*P* value	Beta	SE	*P* value
**Mean SBP**								
	**Study period**							
		Posttest	−1.12	0.90	.21	−1.83	1.18	.12
		Pretest	—^a^	—	—	—	—	—
	**Gender**							
		Female	−.59	0.90	.51	.30	1.11	.79
		Male	—	—	—	—	—	—
	Age (continuous)		.08	0.04	.03^b^	.12	0.04	.01^b^
	**Setting**							
		Rural areas	7.98	0.92	<.01^b^	9.07	1.21	<.01^b^
		Palestinian refugee camps	—	—	—	—	—	—
**Mean DBP**								
	**Study period**							
		Posttest	−.49	0.53	.36	−.90	0.65	.16
		Pretest	—	—	—	—	—	—
	**Gender**							
		Female	−1.89	0.53	<.01^b^	−.73	0.61	.23
		Male	—	—	—	—	—	—
	Age (continuous)		−.02	0.02	.26	−.07	0.02	<.01^b^
	**Setting**							
		Rural areas	1.66	0.54	<.01^b^	2.17	0.67	<.01^b^
		Palestinian refugee camps	—	—	—	—	—	—
**Mean HbA_1c_**								
	**Study period**							
		Posttest	−.87	0.19	<.01^b^	−.22	0.27	.41
		Pretest	—	—	—	—	—	—
	**Gender**							
		Female	−.42	0.17	.01^b^	−.21	0.22	.35
		Male	—	—	—	—	—	—
	Age (continuous)		−.01	0.01	.12	−.03	0.01	<.01^b^
	**Setting**							
		Rural areas	−.65	0.19	<.01^b^	.07	0.25	.76
		Palestinian refugee camps	—	—	—	—	—	—

^a^Refers to reference category.

^b^Refers to statistical significance at .05 CI.

Table 5 summarizes the regression models for mean SBP, mean DBP, and mean HbA_1c_. After controlling for age, gender, and setting, the mean changes in SBP and DBP across the study period were not statistically significant (beta=−1.12 mmHg, SD 0.90, *P*=.21 for mean SBP intervention group; beta=−1.83 mmHg, SD 1.18, *P*=.12 for mean SBP control group; beta=−.49 mmHg, SD 0.53, *P*=.36 for mean for DBP intervention group; and beta=−.90 mmHg, SD 0.65, *P*=.16 for mean DBP control group). Only mean HbA_1c_ retained statistical significance in the multivariate analysis. A mean decrease in HbA_1c_ of 0.87 pretest to posttest period was observed among the intervention group (beta=−.87, SD 0.19, *P*<.01) but not in the control group (*P*=.41). This change was independent of age, gender, and setting. On average, females in the intervention group had a lower HbA_1c_ score compared with their male counterparts (beta=−.42, SD 0.17, *P*=.01). Age was associated with a decrease in HbA_1c_, which was seen in the control group only (beta=−.03, SD 0.01, *P*<.01), and patients in rural areas belonging to the intervention group had a lower HbA_1c_ score as compared with refugee camps (beta=−.65, SD 0.19, *P*<.01).

## Discussion

### Principal Findings

First of its kind in the country, the study revealed promising findings in regard to the use of mobile phone SMS technology to improve the management of NCDs among individuals living in rural areas and Palestinian refugee camps in Lebanon. Postintervention measurements of quality health indicators in the intervention group showed remarkable improvement in comparison with preintervention assessments through enhanced BP control, reduced mean SBP, as well as decrease in HbA_1c_ levels and HbA_1c_ poor control. Comparison across the two settings (ie, rural areas vs refugee camps) showed differential improvements in diabetes and HTN quality indicators where patients living in refugee camps exhibited improved BP control and lower means of SBP and DBP compared with patients from rural areas. Patients in rural areas had comparatively lower HbA_1c_ scores and were at higher odds of conforming to HbA_1c_ testing. No clear direct effect of the implemented mHealth intervention on change in smoking habits, patients’ utilization of PHC services, and compliance with visits for HbA_1c_ testing, eye check-ups, and foot exams were noted.

Our findings of a considerable increase in BP control only in patients receiving the mHealth intervention are in agreement with those of similar studies conducted in Spain, South Korea, and Russia [40,53,54]. In contrast, Wald et al and Orsama et al had findings showing no statistically significant patterns for amelioration neither in BP control nor in means SBP and DBP in the SMS intervention group [55,56]. After controlling for age, gender, and setting, only BP control retained statistical significance in the regression model, with a 28% increase in the odds of BP control in the posttest period as compared with the pretest period. No significant difference for BP control was found across age and gender. There is a paucity of data in the literature with regard to differences in intervention outcomes among hypertensive patients based on age, gender, or setting [40,53,54].

The group that received SMS messages had a clinically important and statistically significant change in glycemic control. More specifically, the intervention group showed a significant decrease both in HbA_1c_ levels (from 8.00%, SD 1.89 to 7.2%, SD 2.06) and in HbA_1c_ poor control (from 28.2%-20.3%) after a 1-year SMS intervention. The control group, on the other hand, showed a slight increase in HbA_1c_ poor control within the same period. The 38% decrease in the odds of HbA_1c_ poor control among the intervention group from the pretest to the posttest study periods was independent of age, gender, and setting in the multivariate analysis. The same applied for the reported change in HbA_1c_ (drop by 0.8%) in the intervention group. This finding concurs with those of a study from Bangladesh, where HbA_1c_ levels dropped by a mean of 0.85% after the SMS intervention [38]. Furthermore, this reported change is higher than those from previous studies, such as a 0.7% reduction in HbA_1c_ posttest in Iraq [57], 0.4% in Saudi Arabia [58], 0.53% in a meta-analysis by Arambepola et al [26], and 0.39% in a meta-analysis by Liu and Ogwu [59]. Other studies have shown a higher reductions in HbA_1c_ posttest ranging from 0.89% to 2.76% [60-63]. In this study, it was shown that HbA_1c_ levels and HbA_1c_ poor control differed significantly across gender, with females in the intervention group having a lower HbA_1c_ score and a lower odds of HbA_1c_ poor control compared with their male counterparts. One explanation relates to females being more attentive to SMS messages [38]. However, such explanation requires confirmation in future studies. On the other hand, in an Iraqi study [57] and a systematic review of 17 articles [64], changes in HbA_1c_ levels following SMS intervention were not related to any of the demographic factors including age, gender, and nationality. Similarly, age was not associated with a decrease in HbA_1c_ in our study’s intervention group, yet the odds of having HbA_1c_ poor control decreased with age for both study groups.

### Difference in Quality Indicators Across Setting

The logistic regression model revealed that participants living in rural areas reported lower odds of BP control compared with patients living in refugee camps in both study groups. This was also apparent in the linear regression model, where the means SBP and DBP were higher among participants in rural areas as compared with their refugee camps counterparts. In contrast, patients living in rural areas had a lower HbA_1c_ score in intervention group and were also at higher odds of conforming to HbA_1c_ testing guidelines in both study groups as compared with Palestinian refugee camps participants. The fact that the improvement in diabetes and HTN quality indicators do not align across the two settings flags an improvement opportunity in the mHealth program design and in the NCD care configurations across the two settings. It also highlights an opportunity for learning across the two settings. The fact that BP control was better among patients living in refugee camps in both study groups might reflect the role of the UNRWA’s NCD program integrated in the intervention and control centers and entailing primary, secondary, and tertiary NCDs’ prevention [10]. The presence of such a program at UNRWA may have enhanced the effectiveness of the mHealth interventions and facilitated improved access to services. However, as our study reveals, further efforts are needed to improve diabetes prevention and care for Palestinian refugees in regards to improving their glycemic control and treatment compliance [65]. Furthermore, the finding that patients’ conforming to HbA_1c_ testing guidelines was better among patients from rural areas in both study groups echoes that the MOPH NCD program was more effective in diabetes management and control as compared with HTN [66]. The presence of an advanced diabetes control program may have enhanced the effectiveness of the mHealth interventions employed in this study. Our study reveals that the success and effectiveness of mHealth interventions is contingent on the service configurations and care programs employed at PHCCs. Despite the best efforts of the research team to ensure comparable service configuration at both rural PHCCs and those at UNRWA refugee camps, it appears that the maturity of the existing programs and the service configurations at each setting played an important role in the care outcomes at the end of the study. This underlines the importance of employing integrative approaches of diseases prevention and control in which existing NCD programs in underserved communities (ie, rural and refugee camps settings) are coupled with innovative approaches such as mHealth to provide an amplified effect of traditional NCD-targeted care.

### Changes in Smoking Habits

Our study revealed an unremarkable and insignificant reduction in the proportion of smokers in intervention and control groups after a 1-year SMS intervention. This is in agreement with a study that showed that lifestyle behaviors such as smoking were not modified throughout the self-care education intervention whether via SMS, pamphlets or face-to-face meetings [36]. However, our finding is in contrast with other studies carried out in Iran, United Kingdom, and New Zealand [63,67,68], the three of which revealed a significant positive modification in smoking cessation in the intervention group based on SMSs. mHealth has the potential to support smoking cessation, especially when considered as an add-on to other smoking cessation services [67,69]. This again reveals that a successful mHealth program is contingent on the presence of other programs to support the creation of successful and sustainable change in behavior. Yet, it is worth mentioning that achieving actual changes in smoking habits and attaining smoking cessation necessitate employing integrated theoretical models of health behavioral change to design effective health behavior interventions. Such models recognize that behavioral change is dependent on a number of factors that are necessary for it to take place [70]. These factors include the individual’s strong intention to perform this behavior; having necessary information, skills, and capabilities required to actually perform it; and is not faced by any environmental constraints that may hinder the behavioral performance [70]. Although our mHealth intervention may have supported one of these factors toward the achievement of changes in smoking habits, it may have not interfered with other factors that are necessary to address to witness a significant and sustainable change.

### Changes in Patients’ Utilization of Primary Care Services

In this study, targeted SMSs for reminders did not generate a clear intervention effect on patients’ utilization of primary care services, as well as their compliance with visits for HbA_1c_ testing, eye check-ups, and foot exams. Both bivariate and multivariate analyses revealed that access for annual HbA_1c_ testing increased significantly in both the intervention and control groups. This can be explained by the initiatives of the MOPH and UNRWA’s NCD programs aiming at the early detection of NCDs, proper management of these diseases, and the promotion of health awareness in all MOPH and UNRWA centers. However, the results of this study’s QIs reveal that these efforts were translated into measurement but not into outcomes, except for the intervention group where patients were receiving SMS messages. Furthermore, intervention bias could have taken place because QI collectors at PHCCs were aware of data collection post intervention. As a matter of fact, the increased percentage of recorded dates of visits to PHCCs for HbA_1c_ testing in both the control and intervention groups may be the result of improved documentation rather than an actual enhanced access to PHC services. Nonetheless, clinical measurements of QIs, such as HbA_1c_ levels, as well as SBP and DBP levels, reflect the general centers’ performance as they represent actual results rather than recorded dates. In addition, age was found to be statistically associated with annual HbA_1c_ testing for the intervention group, reflecting a better compliance. Similarly, in New Jersey, older age seemed to have contributed to enhanced access to health services as older individuals may have conditions which necessitate a closer follow-up in PHCCs [71].

Studies have shown that SMS reminders considerably ameliorate the prospect of attending clinical appointments in general [33,72,73]. However, there is a notable gap in the literature with regard to whether SMS reminders sent to patients’ mobiles are successful and effective in decreasing nonattendance and increasing compliance to eye check-ups and foot exams among diabetic and hypertensive patients. In this study, there was a decrease in the access for annual eye check-up and foot exam among patients in both groups, in the posttest as compared with the pretest. This decrease was significant only for annual eye check-up among both hypertensive and diabetic patients. Intervention and control PHCCs assured that the same ophthalmologists are still providing eye care in these centers, and none of them quitted. Yet, the decrease in the access for annual eye check-up and foot exam can be explained by the considerable strain that the Syrian refugee influx in Lebanon is putting on host communities and on the resources in PHCCs. Consequently, the demand for chronic diseases’ care has increased dramatically leading to long waiting times in PHCCs and causing providers to practice in more than one center. Hence, Lebanese patients are adversely affected by an increased competition for accessing services. Although this was less evident in the general care for chronic diseases because of the presence of a good number of providers, competition for services was more pronounced in referral to specialist care as in the case of ophthalmologists as their numbers in rural and refugee camps is quite limited.

Additional efforts should be made from the providers’ side to underscore the importance of improved patients’ compliance with visits for HbA_1c_ testing, eye check-ups, and foot exams. Providers are encouraged to utilize the integrated online modules, one of this study’s provider-side eHealth tools, especially the provider-patient communication strategies to focus on the importance of increasing compliance. Conveying face-to-face healthy information has a beneficial effect on adherence. Therefore, using SMSs as a reminder can come after face-to-face education to support it [36,42].

### Limitations

A number of limitations in this study are worth mentioning. Data on age and gender of certain participants in both the intervention and control groups were missing. In addition, the study is characterized by its large sample size, which may have led to statistical significance without necessarily a parallel clinical or practical significance. Our results cannot be solely attributed to our intervention; the presence of advanced NCD programs at both the MOPH and UNRWA PHCC networks may have biased the findings, especially in the cases where a control site showed a significant change. Given that in some cases the owners of the phone numbers to which the SMSs were sent were not the patients themselves but rather family members, the interventional SMS messages may have not been transmitted to their final recipients (ie, patients) who are the target population of our study.

Other limitations may be embedded in the design of the intervention itself. For example, patients of low literacy level may have not benefitted optimally from the intervention because of a decreased capacity of understanding its content. Thus, it is worth bringing to attention for future research the need for pilot testing the SMS text messages interventions with individuals of low literacy level, using further simplified content of messages, and more importantly coupling SMS text messages with voice messages to enhance equitable access of illiterate patients to the information shared [74].

### Policy and Practice Recommendations

Findings from this study should be considered by decision makers at the MOPH and the UNRWA, as the employed eHealth strategies, especially SMS messages, could be easily implemented within the PHC context and adapted to suit all diabetic and hypertensive patients across all centers. Given that the proposed eHealth interventions stem from the needs of the communities served, decision makers are advised to scale-up and use mHealth as a strategy for improving access to PHC, especially with the ongoing influx of Syrian refugees. However, decision makers are reminded to ensure the presence of adequate NCD curative and preventive services to enhance the effectiveness of mHealth programs. The study can also serve as a guidance in the formulation of the national eHealth policy in Lebanon. The findings should also be of interest to primary care providers, who should be adequately trained on accessing the proposed eHealth tools, while emphasizing to patients the importance of SMSs as a method to increase awareness about diseases and compliance to treatment. As mHealth is promising in delivering messages to hard-to-reach populations, the findings of this study may be applicable to similar contexts in the region.

### Conclusions

Given the potential benefits of mHealth, more specifically SMS-based health interventions for the management and control of chronic conditions, its implementation in the EMR, and specifically Lebanon, is crucial. In this study, the statistically significant improvements in clinical measurements of NCD-related QIs among diabetic and hypertensive patients in Lebanese rural areas and Palestinian refugee camps reveal that the employment of SMSs may make a difference in NCDs care. The most pronounced effect was observed in improved BP control, mean SBP, HbA_1c_ poor control, and mean HbA_1c_ among patients who received weekly SMSs for 1 year. Further studies are needed to provide concrete evidence with regard to the effectiveness and usefulness of reminder SMSs for improving compliance and access to services in PHC centers. Future research is also advisable on patients’ perceptions and views on the acceptability and utility of the SMS service, as well as providers’ attitudes and barriers toward the full implementation of clinical guidelines. A separate analysis of different activities using this project’s eHealth interventions is currently underway with promising results. mHealth is a simple and a socially acceptable technique that can be integrated into routine care at a low cost. As mobile phones are widely accessible across the globe, including hard to reach and underserved populations, the need is underscored for additional studies to provide evidence on the utility of SMS-based health interventions and their impact on the care of underserved individuals and communities.

## Abbreviations

BP: blood pressure
DBP: diastolic blood pressure
eHealth: electronic health
EMR: Eastern Mediterranean Region
HbA _1c_: glycated hemoglobin
HTN: hypertension
mHealth: mobile health
MOPH: Ministry of Public Health
LMIC: low- and middle-income countries
NCD: noncommunicable disease
OR: odds ratio
PHC: primary health care
PHCC: primary health care center
QI: quality indicator
SBP: systolic blood pressure
SMS: short message service
UNRWA: United Nations Relief and Works Agency

## Appendix

Multimedia Appendix 1 CONSORT‐EHEALTH checklist (V 1.6.1).
